# Supplementary material for: Global meta-analysis shows reduced quality of food crops under inadequate animal pollination
Source: Nat Commun. 2023 Jul 25;14:4463. doi: 10.1038/s41467-023-40231-y (PMC10368628; doi:10.1038/s41467-023-40231-y)
Supplement: Supplementary file 1 — Supplementary Information [file 41467_2023_40231_MOESM1_ESM.pdf]

Supplementary Information

Global meta-analysis shows reduced quality of food crops under inadequate animal pollination

Authors:

Elena Gazzea[1]*, Péter Batáry[2], Lorenzo Marini[1]

Affiliations:

[1] Department of Agronomy, Food, Natural resources, Animals and Environment (DAFNAE), University of Padua, Legnaro (Padua), Italy

[2] "Lendület" Landscape and Conservation Ecology, Institute of Ecology and Botany, Centre for Ecological Research, Vácrátót, Hungary

* Corresponding author, e-mail: elena.gazzea@unipd.it

Contents:

Supplementary Figures 2
Supplementary Tables 14
Supplementary Note 23
Supplementary References 24

Supplementary Figures

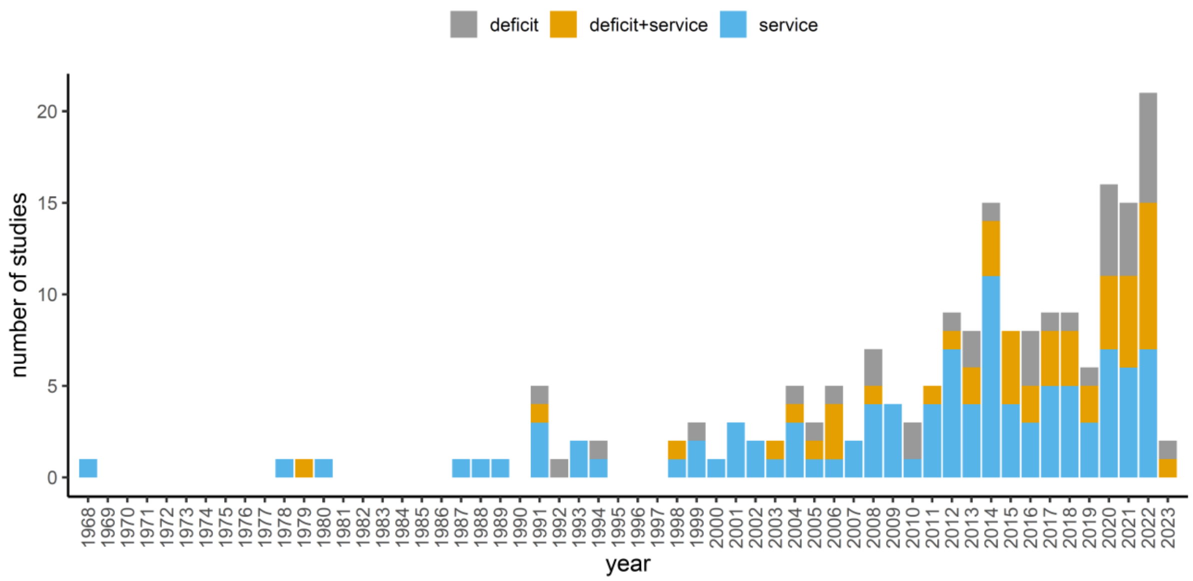

Supplementary Fig. 1 – Number of studies across years. The figure shows the number of studies included in the meta-analysis per publication year and per pollination metric.

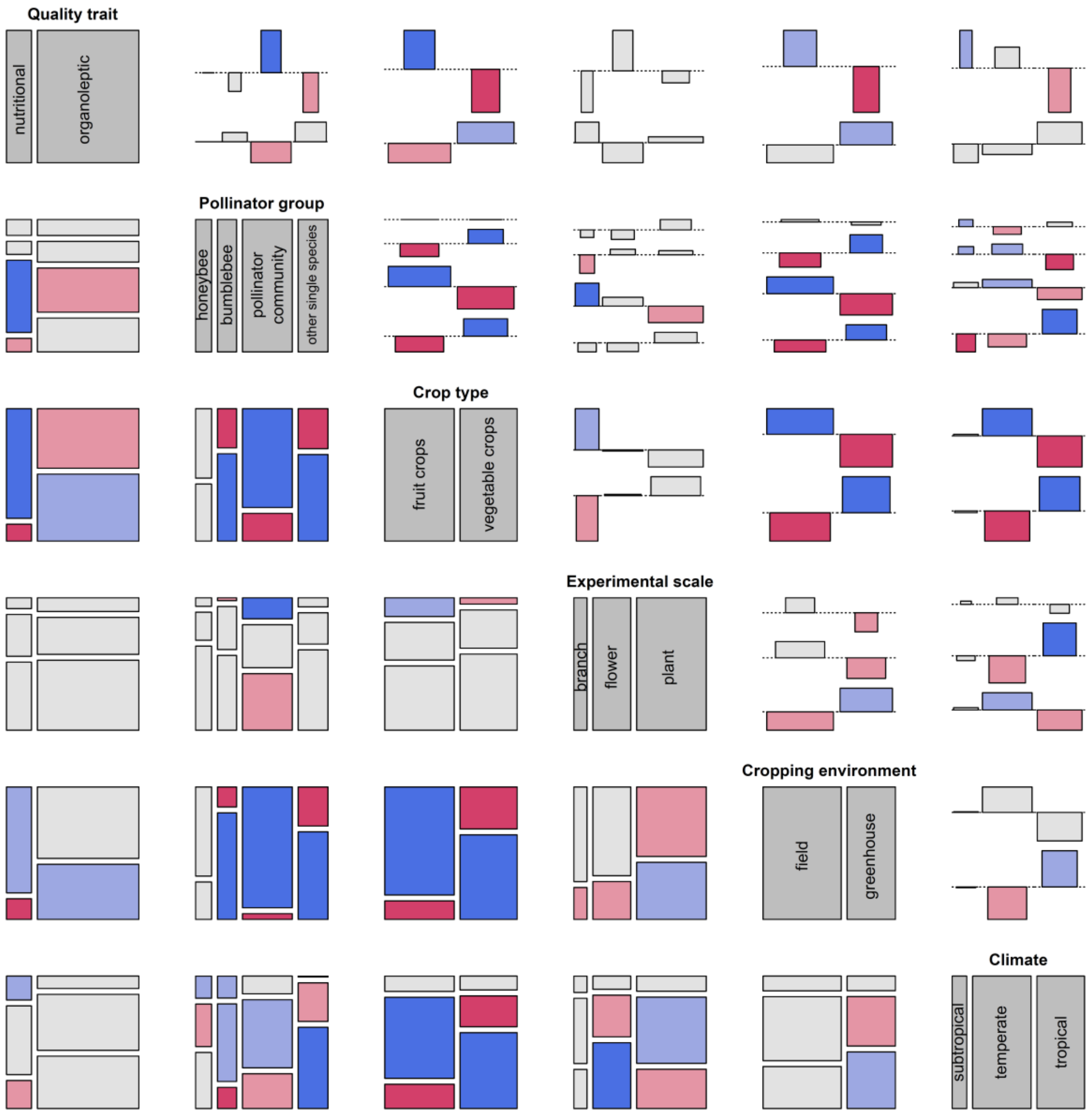

Supplementary Fig. 2 – Matrix of mosaic plots showing the association among categorical moderators used in the pollination service meta-analysis. Mosaic plots are displayed in the lower triangle matrix, with tiles proportional to the observed frequencies in the levels of each moderator. Association plots are displayed in the upper triangle matrix, with shaded bars proportional to the residuals (deviations from independence) and indicating the strength of the association between different levels of the moderators. Colours follow default coding: white for residuals with an absolute value smaller than 2; light blue and red for positive and negative residuals exceeding an absolute value of 2, and full saturation for residuals exceeding an absolute value of 4. The plots help visualising the potential collinearity among moderators. To improve figure clarity, only fruit crops and vegetable crops are displayed within the crop type moderator.

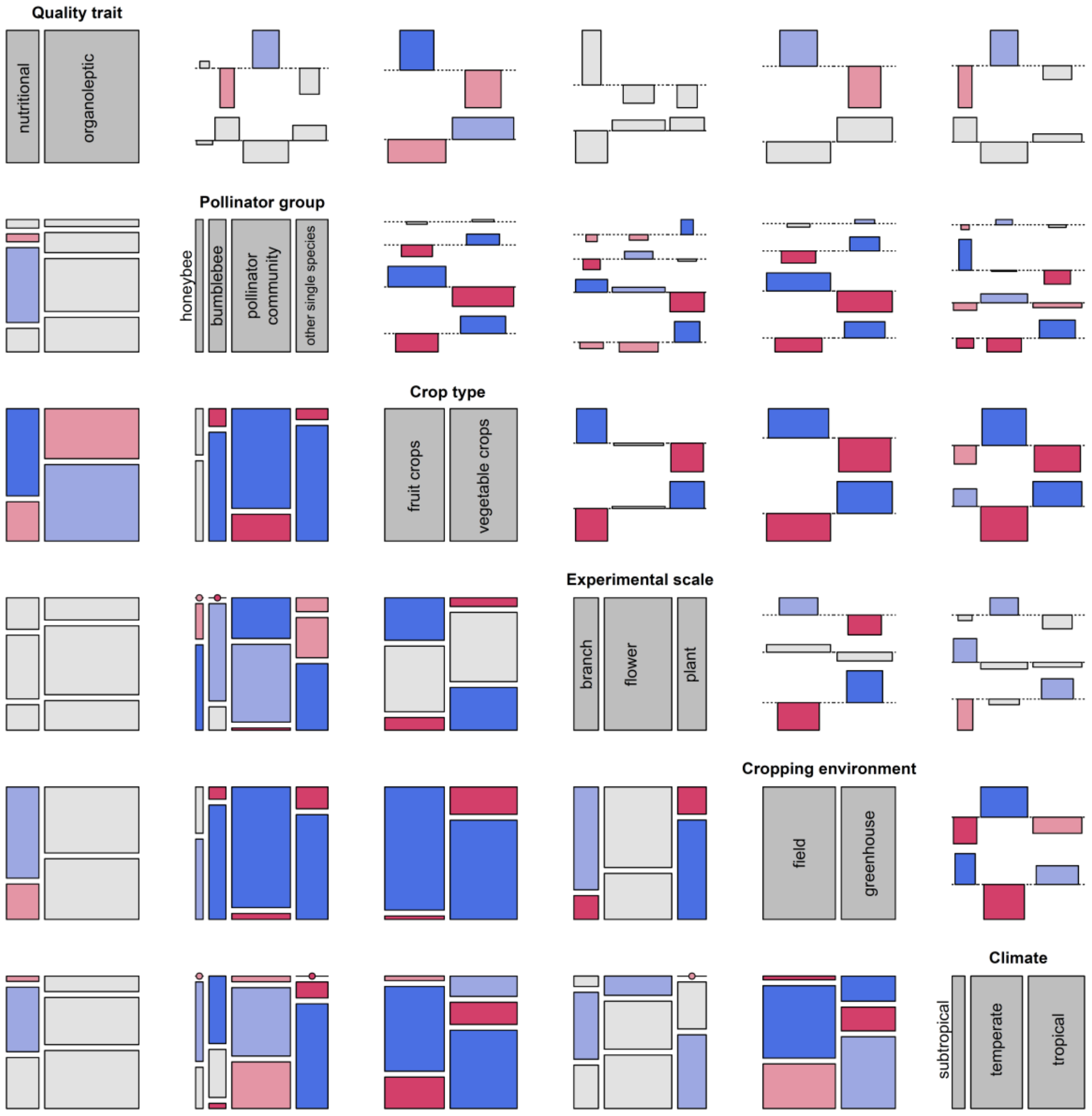

Supplementary Fig. 3 – Matrix of mosaic plots showing the association among categorical moderators used in the pollination deficit meta-analysis. Mosaic plots are displayed in the lower triangle matrix, with tiles proportional to the observed frequencies in the levels of each moderator. Association plots are displayed in the upper triangle matrix, with shaded bars proportional to the residuals (deviations from independence) and indicating the strength of the association between different levels of the moderators. Colours follow default coding: white for residuals with an absolute value smaller than 2; light blue and red for positive and negative residuals exceeding an absolute value of 2, and full saturation for residuals exceeding an absolute value of 4. The plots help visualising the potential collinearity among moderators. To improve figure clarity, only fruit crops and vegetable crops are displayed within the crop type moderator.

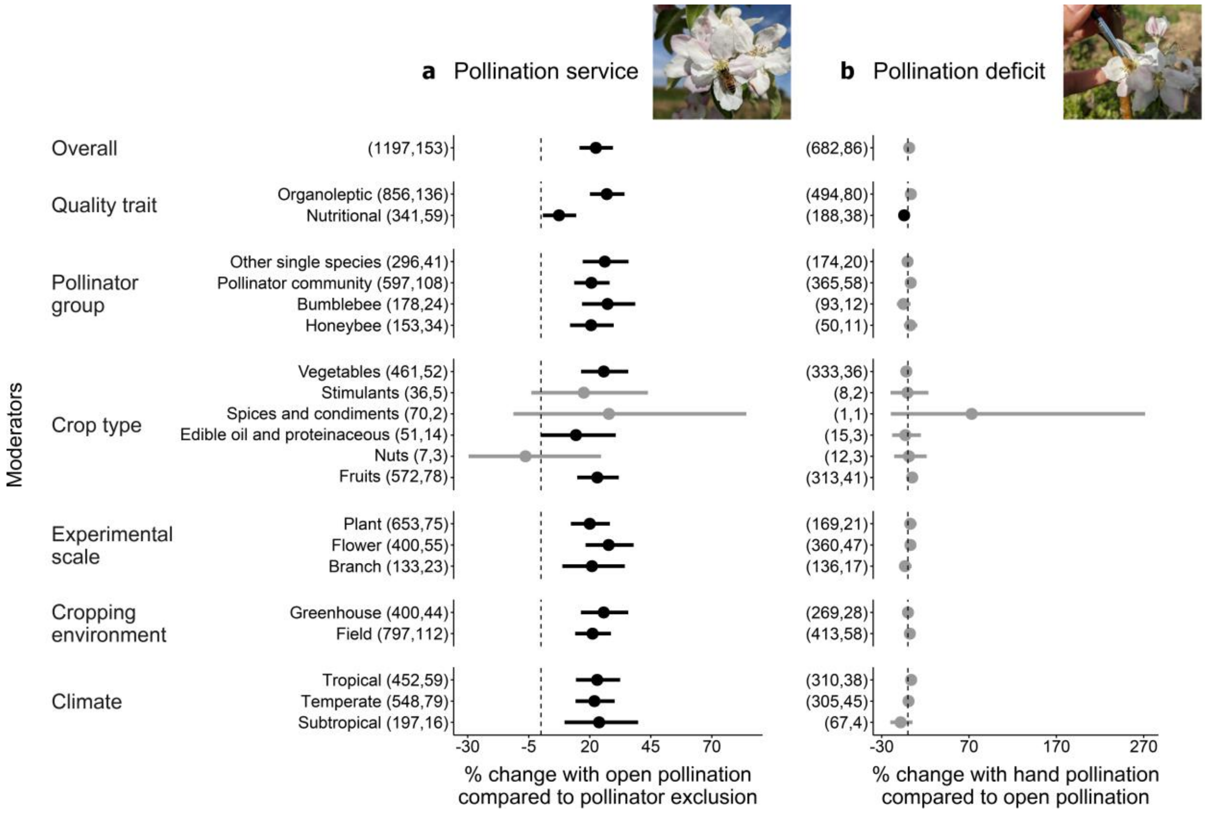

Supplementary Fig. 4 – Change in food crop quality for different moderators used in the pollination service (a) (k=1197 effect sizes) and pollination deficit (b) (k=682 effect sizes) datasets. Bars around the means indicate 95% CI. Bars not intersecting with zero (dashed line) are coloured in black and indicate a statistically significant change. Estimates are derived from models without the intercept to test within-group differences. The overall pollination outcome was estimated as the pooled effect size (null model). The first and second numbers in parentheses following each moderator level name indicate, respectively, the number of effect sizes used to derive the displayed statistics, and the number of studies included in each calculation. Figure highlights non-significant effect sizes for spices and condiments. For clearer visualisation of mean values and confidence intervals of all other moderator levels, refer to Figure 3 in the main text.

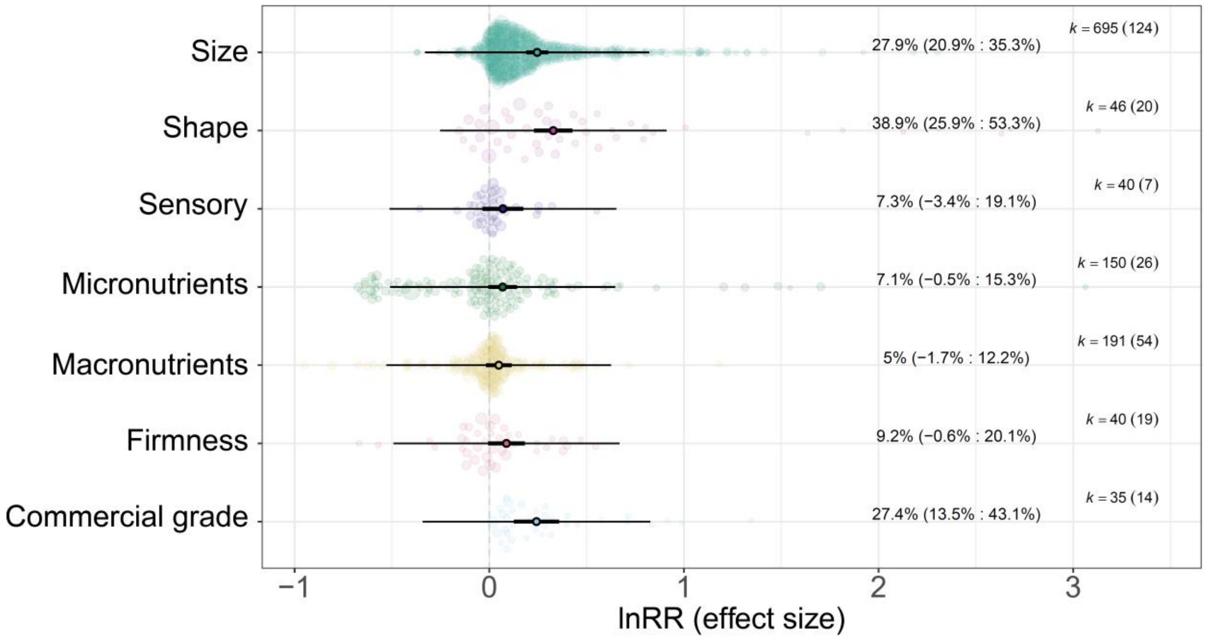

Supplementary Fig. 5 – Orchard plot of quality traits for pollination service (k=1197 effect sizes). The line in bold represents the 95% confidence interval, the thin line represents the 95% prediction interval. *k*=number of effect sizes used to derive the displayed statistics. Numbers in parentheses following *k* indicate the number of studies included in calculations. The size of the bubbles is proportional to the inverse measure of the standard error, thus the larger the bubble the greater the precision of the effect size. If the confidence bar falls in the positive side and does not intersect with zero, we interpret that pollination affects quality positively. Percentage numbers indicate the converted mean values (and confidence intervals) as percentage increase over pollinator exclusion treatment. Orchard plot was produced using orchaRd 2.0 package [1].

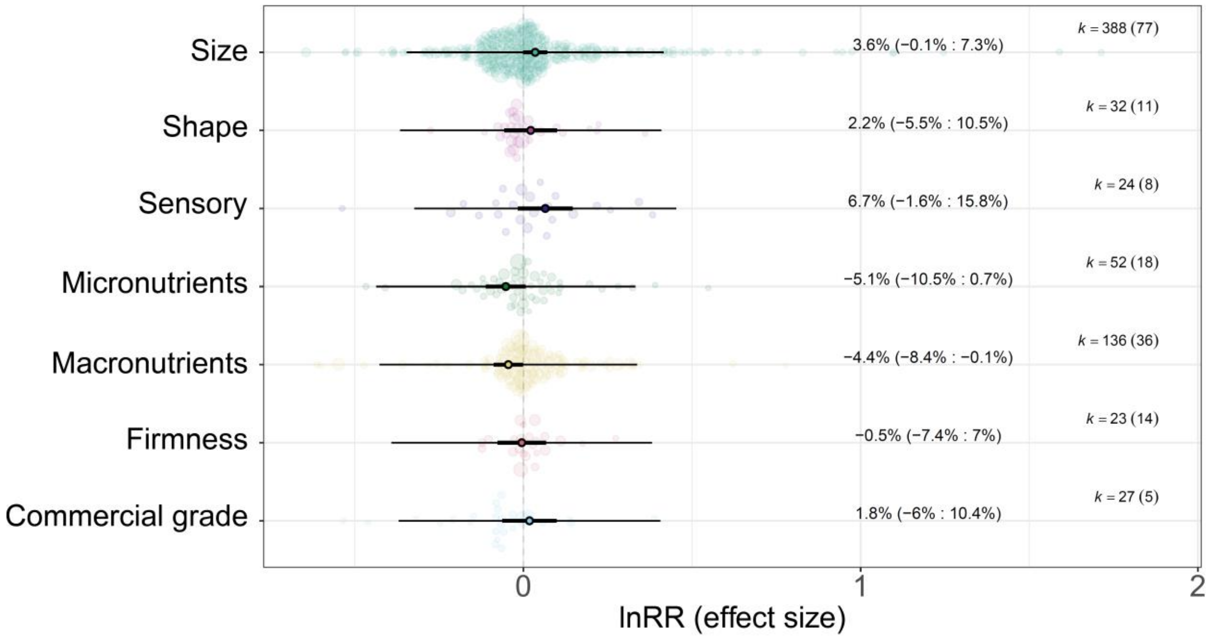

Supplementary Fig. 6 – Orchard plot of quality traits for pollination deficit (k=682 effect sizes). The line in bold represents the 95% confidence interval, the thin line represents the 95% prediction interval. *k*=number of effect sizes used to derive the displayed statistics. Numbers in parentheses following *k* indicate the number of studies included in calculations. The size of the bubbles is proportional to the inverse measure of the standard error, thus the larger the bubble the greater the precision of the effect size. If the confidence bar falls in the positive side and does not intersect with zero, we interpret that pollination affects quality positively. Percentage numbers indicate the converted mean values (and confidence intervals) as percentage increase over open pollination treatment. Orchard plot was produced using orchaRd 2.0 package [1].

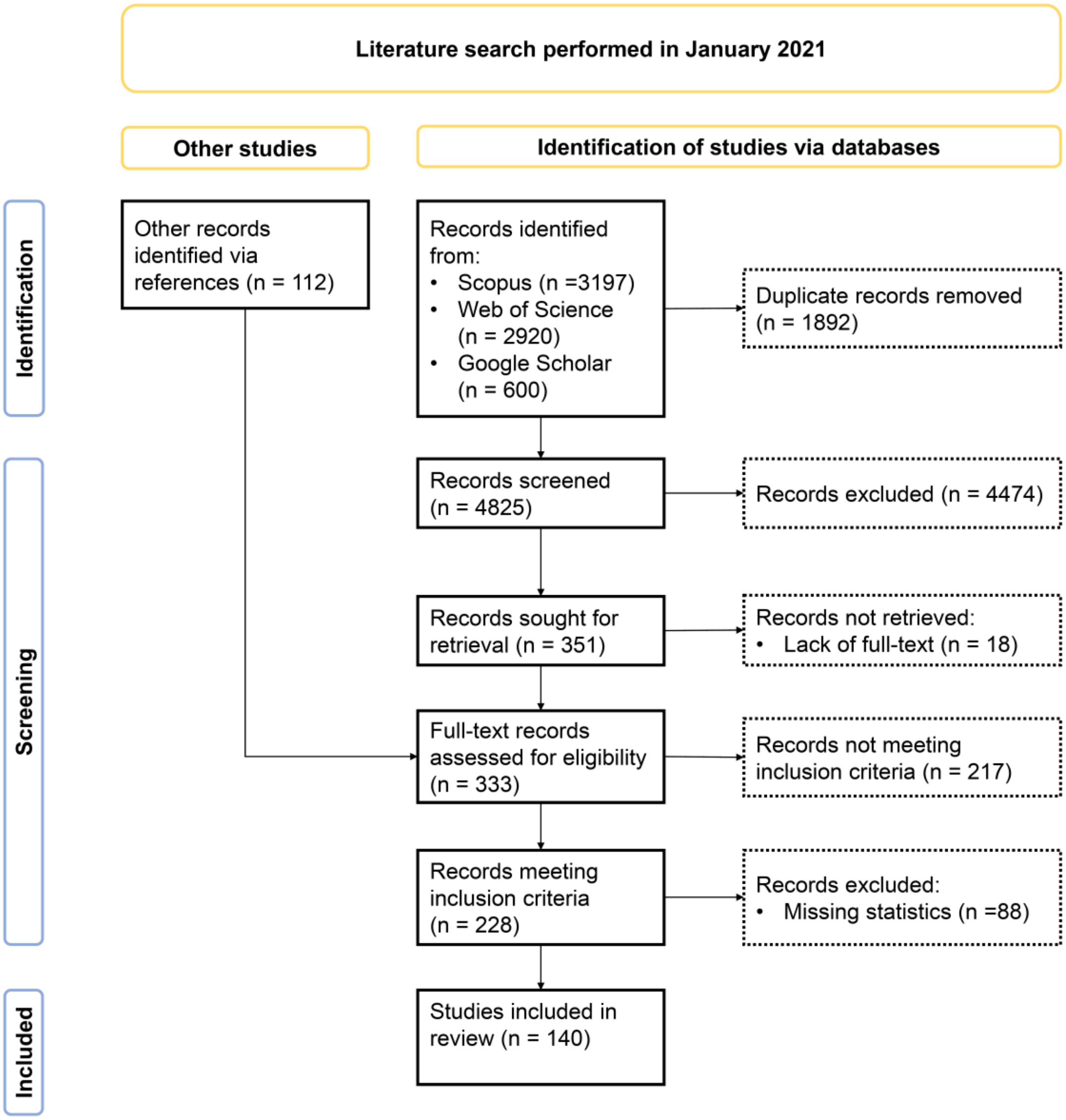

Supplementary Fig. 7 – Modified PRISMA flow diagram. A flowchart showing the number of studies included and excluded at each stage of an initial version of the literature review conducted on the 28th of January 2021. For the full search string refer to Methods in the main text.

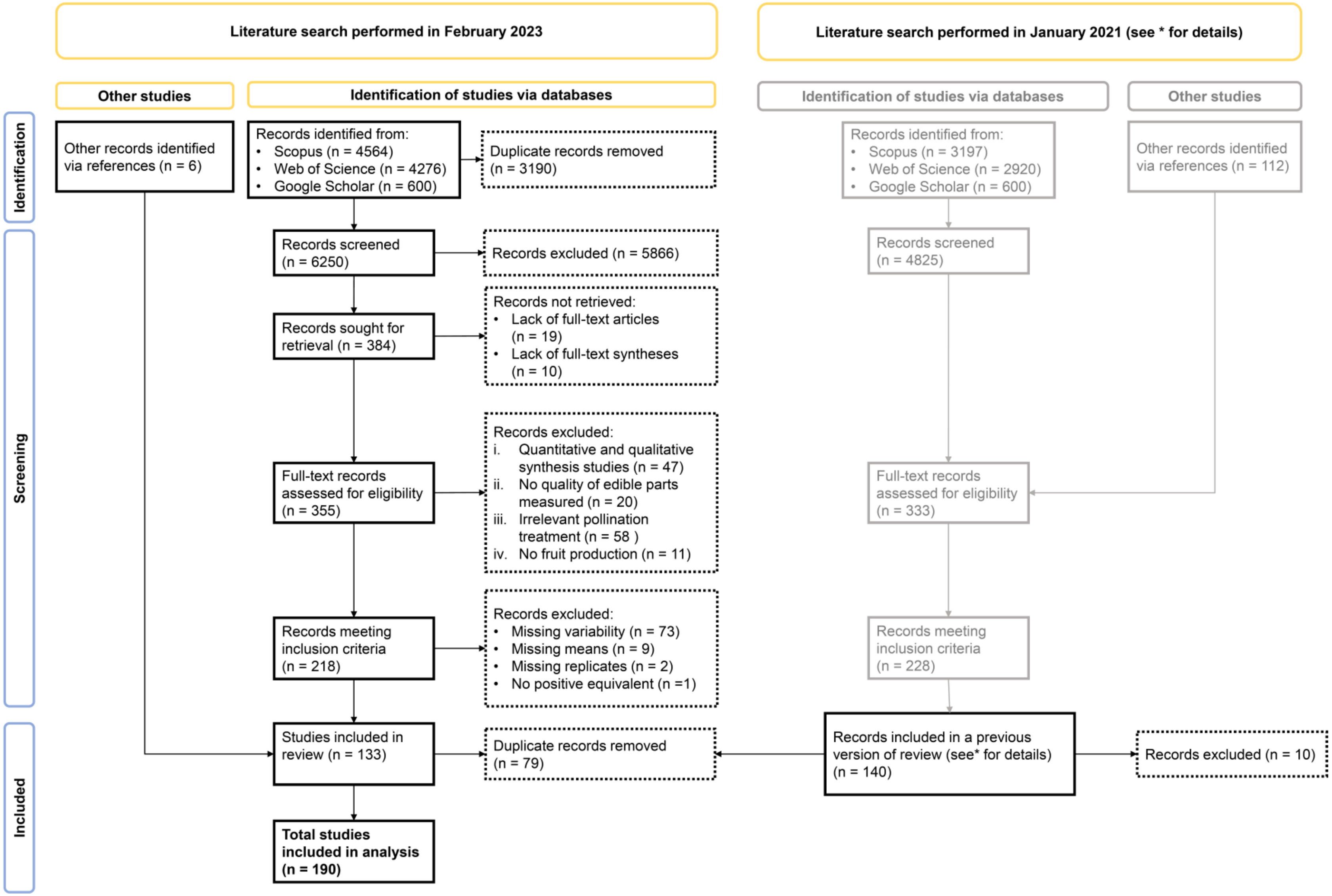

Supplementary Fig. 8 – Modified PRISMA flow diagram. A flowchart showing the number of studies included and excluded at each stage of the literature search conducted on the 23rd of February 2023 (Scopus and WoS) and on the 28th of February 2023 (Google Scholar). For the full search string refer to Methods in the main text. *The studies used in the meta-analysis were supplemented with an initial version of the literature review conducted in January 2021. For the full PRISMA diagram refer to Supplementary Fig. 7, and for the full search string refer to Methods in the main text.

a

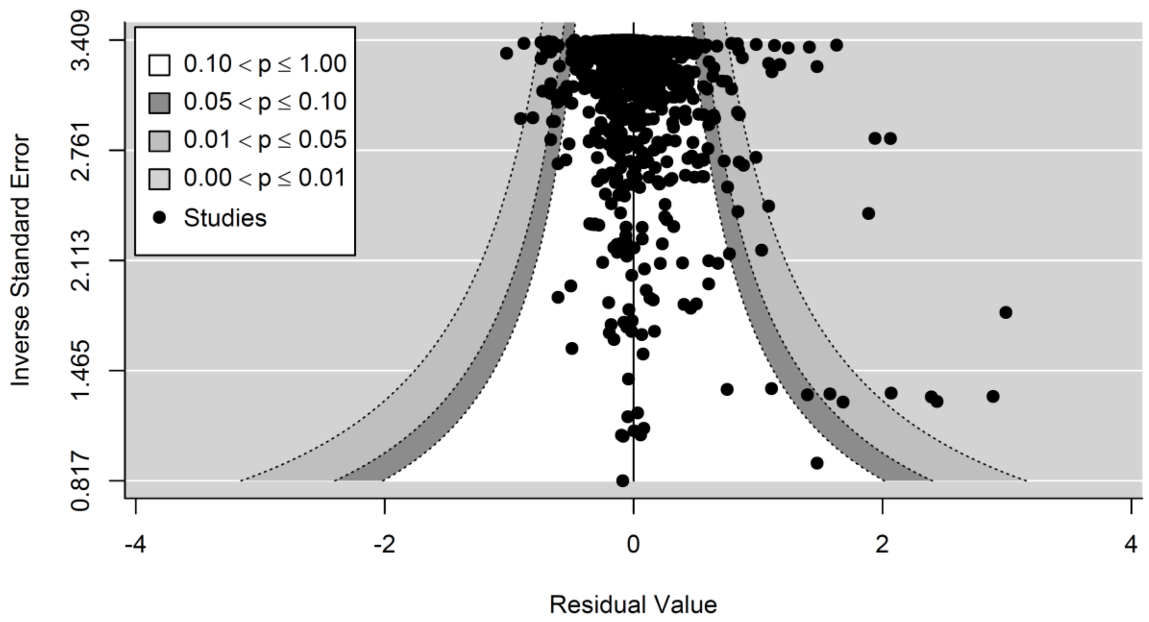

b

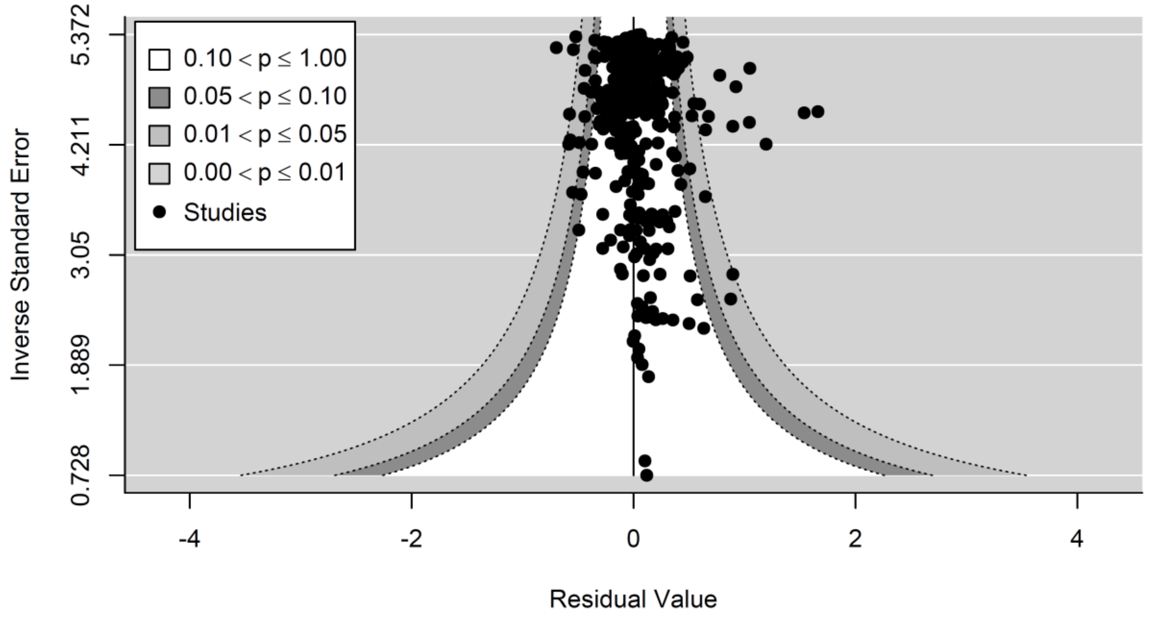

Supplementary Fig. 9 – Contour-enhanced funnel plots of the residuals from the models including all significant moderators for pollination service (a) and pollination deficit (b). More precise studies are located at the top of the plot and less precise studies are located at the bottom. These contour-enhanced funnel plots display areas of (two-sided) statistical significance [2].

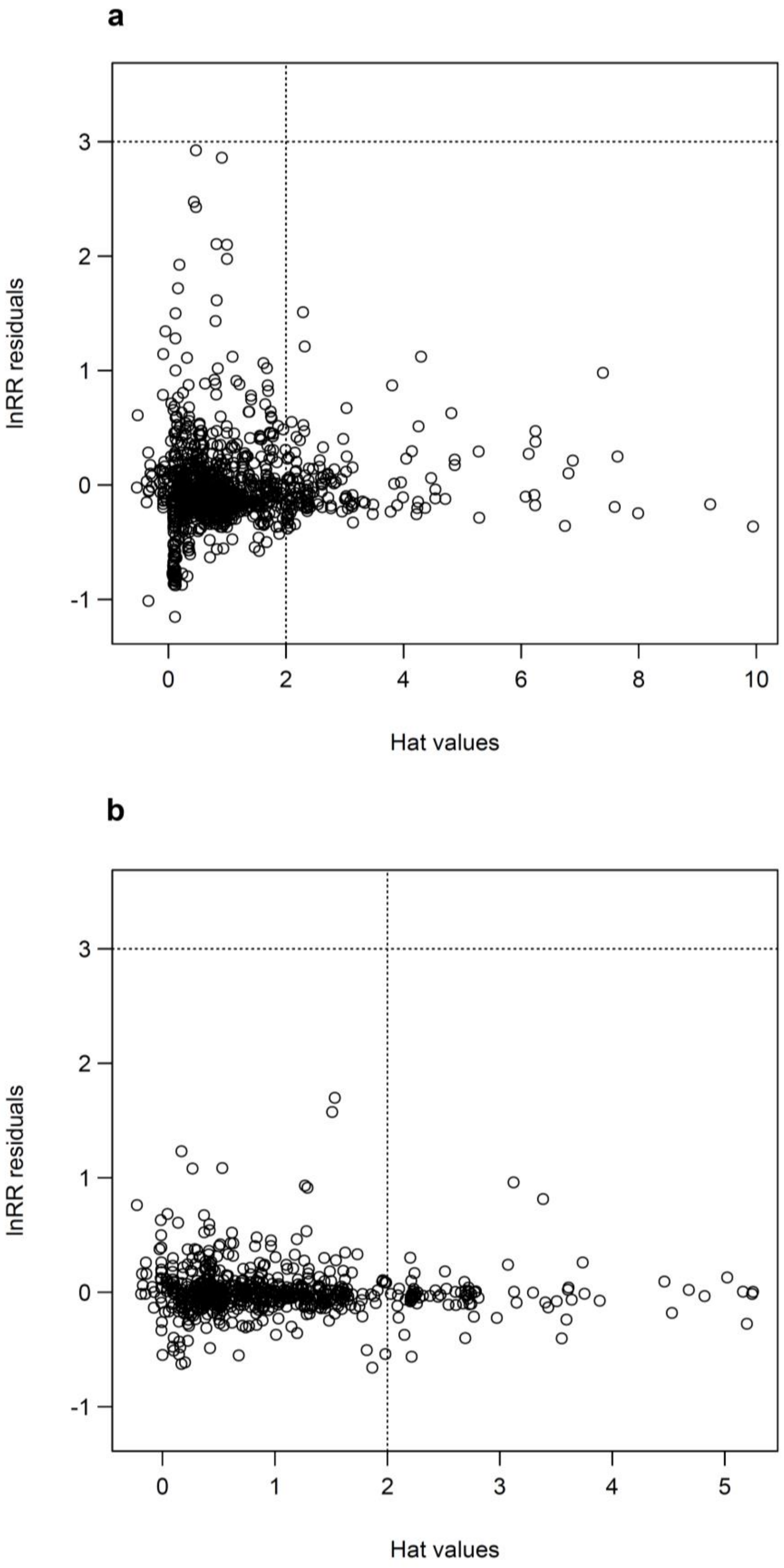

Supplementary Fig. 10 – Hat values and respective internally standardized residuals for each effect size in the pollination service dataset (a) and the pollination deficit dataset (b). Dotted lines indicate the thresholds above which observations are considered influential outliers.

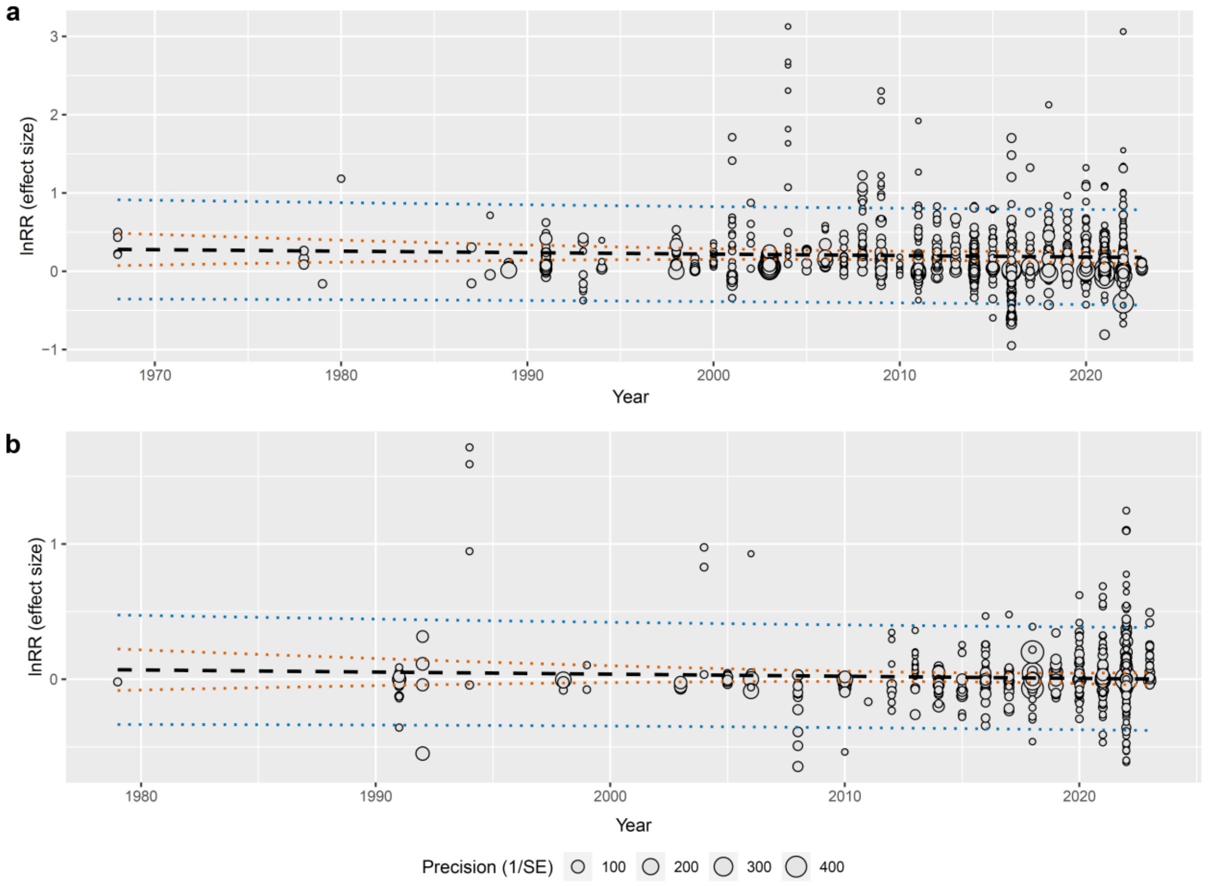

Supplementary Fig. 11 – Time lag bias in the pollination service (a) and the pollination deficit dataset (b). Red dotted lines indicate 95% confidence intervals and blue dotted lines indicate 95% prediction intervals.

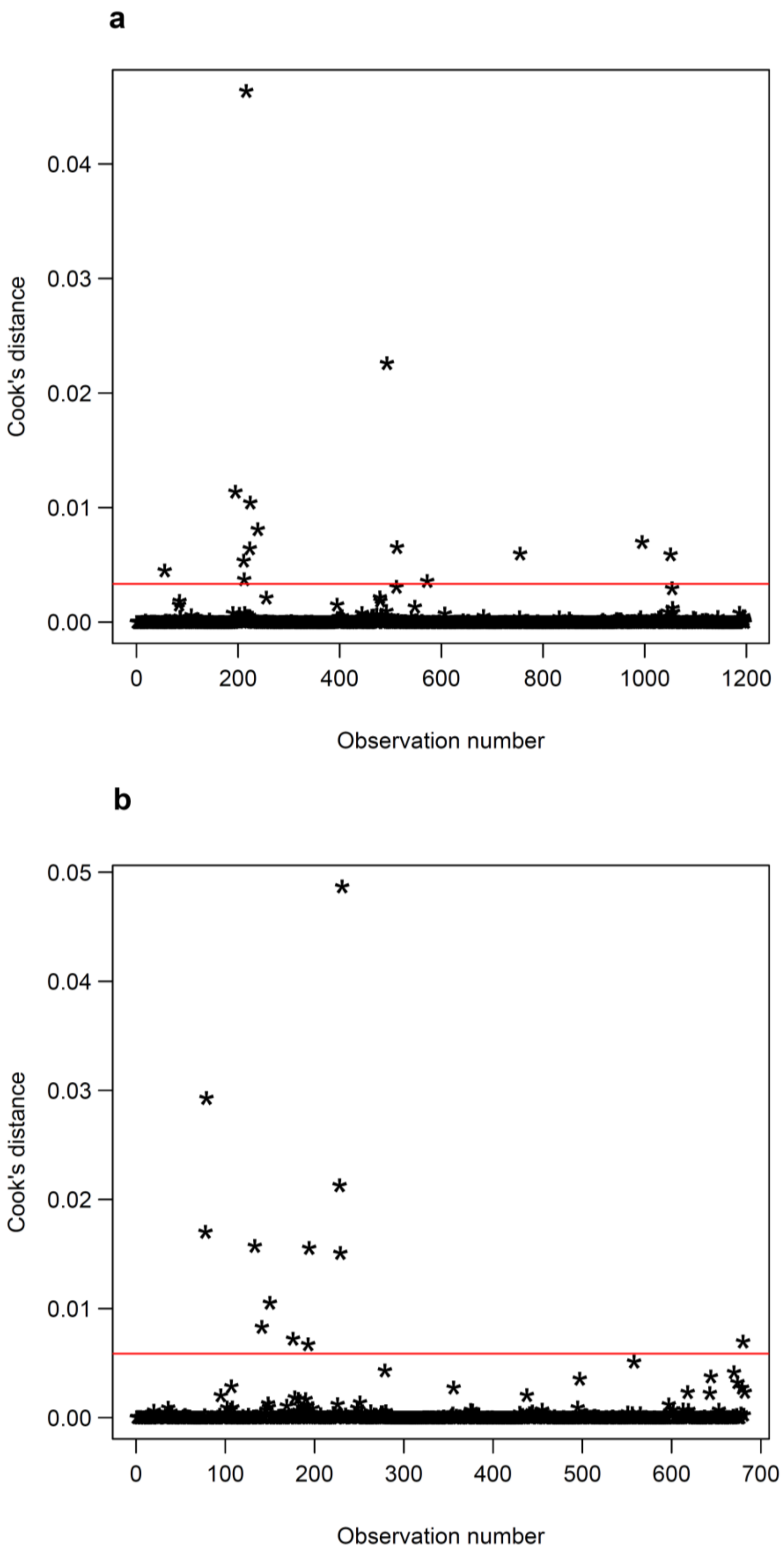

Supplementary Fig. 12 – Cook's distance of each effect size for pollination service (a), and deficit (b). Red lines indicate the thresholds for considering an observation as influential. The method was used to identify the most influential observations.

Supplementary Tables

Supplementary Table 1 – List of crops investigated in the studies included in our meta-analysis. The table specifies the categories of crops used in the analyses, the number of effect sizes per individual crop and per pollination metric, and the main pollinator groups of each crop according to Klein et al. [3].

Categories of crops used in the meta-analysis	Original crops measured in the single studies	N effect sizes pollination deficit	N effect sizes pollination service	Main pollinator groups
Fruit crops	Apple	79	82	Insects
	Blueberry	37	22	Insects
	Cape gooseberry	8	8	Unknown
	Cranberry	0	2	Insects
	Custard apple	1	0	Insects
	Date	22	0	Unknown
	Fig opuntia	0	6	Insects
	Grape	9	15	Insects
	Honeyberry	3	9	Unknown
	Jackfruit	2	0	Insects
	Kiwifruit	11	43	Insects
	Loquat	5	39	Insects
	Lulo	4	0	Insects
	Mandarin	0	7	Insects
	Mango	0	10	Insects
	Maracuja	14	0	Insects, birds
	Orange	0	4	Insects
	Peach	0	18	Insects
	Pear	14	30	Insects
	Pitaya	31	34	Unknown
	Plum	32	14	Insects
	Rambutan	0	4	Insects
	Raspberry	0	11	Insects
	Sonneratia alba	4	4	Unknown
	Strawberry	32	209	Insects
	Sweet cherry	1	1	Insects
	Tangelo	4	0	Insects
Nut crops	Almond	3	6	Insects
	Macadamia	9	1	Insects
Oil and proteinaceous crops	Field bean	0	1	Insects
	Lupin	0	10	Insects
	Mustard	4	2	Insects
	Paeonia ostii	7	13	Unknown
	Rapeseed	0	16	Insects
	Sunflower	4	9	Insects
Spices and condiments	Fennel	1	70	Insects

Stimulant crops	Coffee	8	36	Insects
Vegetable crops	Bitter melon	6	0	Insects
	Cowpea	0	6	Insects
	Cucumber	47	52	Insects
	Eggplant	25	63	Insects
	Melon	75	53	Insects
	Okra	0	12	Insects
	Pepper	24	128	Insects
	Pumpkin/Squash	19	2	Insects
	Tomato	118	141	Insects
	Watermelon	19	0	Insects
	Zucchini	0	4	Insects

Supplementary Table 2 – List of pollinator taxa investigated in the studies included in our meta-analysis. The table specifies the pollinator groups used in the analyses and the number of effect sizes per pollinator taxa and per pollination metric.

Pollinator groups used in the meta-analysis	Species	N effect sizes pollination deficit	N effect sizes pollination service
Honeybee	*Apis cerana*	2	12
	Apis cerana indica	0	7
	Apis florea	0	1
	Apis mellifera	48	133
Bumblebee	*Bombus atratus*	0	12
	Bombus ephippiatus	0	5
	Bombus haemorrhoidalis	0	11
	Bombus hypocrita	0	6
	Bombus impatiens	9	16
	Bombus lantschouensis	7	0
	Bombus terrestris	71	122
	Bombus vosnesenskii	6	6
Pollinator community	Pollinator community	365	597
Other single species	*Amegilla holmesi*	3	3
	Amegilla murrayensis	4	0
	Apis cerana indica + Bombus haemorrhoidalis	0	6
	Augochloropsis sp.	0	2
	Austroplebeia australis	0	9
	Bombus terrestris + Apis mellifera	0	7
	Calliphora spp.	0	2
	Chrysoperla carnea	0	6
	Dactylurina schmidti	12	0
	Episyrphus balteatus	5	5
	Eristalinus aeneus	0	6
	Euglossa sp.	4	4
	Eupeodes corollae	0	8
	Eupeodes latifasciatus	5	5
	Exomalopsis sp.	0	2
	Geniotrigona thoracica	9	9
	Heterotrigona itama	32	36
	Hoverfly species	5	5
	Hypotrigona gribodoi	8	5
	Hypotrigona ruspolii	0	4
	Lucilia sericata	0	2
	Melipona bicolor	8	8
	Melipona bicolor + Nannotrigona testaceicornis	4	4
	Melipona fasciculata	1	1
	Melipona quadrifasciata	3	21
	Melipona quadrifasciata anthidioides	0	4

	Melipona scutellaris	0	4
	Melipona subnitida	3	3
	Meliponula bocandei	12	0
	Meliponula ferruginea	8	0
	Meliponula lendliana	8	0
	Meliponula togoensis	8	0
	Nannotrigona perilampoides	8	4
	Nannotrigona testaceicornis	8	17
	Nannotrigona testaceicornis + Tetragonisca angustula + Apis mellifera	0	3
	Osmia aglaia	0	1
	Osmia cornuta	0	6
	Osmia cornuta + Lucilia sericata	0	2
	Paratrigona lineata	0	5
	Paratrigona sp.	4	4
	Peponapis pruinosa	1	2
	Plebeina hildebrandti	5	0
	Scaptotrigona depilis	0	9
	Scaptotrigona mexicana + Tetragonisca angustula	0	2
	Sphaerophoria rueppellii	0	8
	Tetragonisca angustula	0	1
	Tetragonula clypearis	0	4
	Tetragonula laeviceps	0	4
	Trigona carbonaria	0	9
	Trigona iridipennis	0	2
	Trigona laeviceps	0	2
	Trigona minangkaba	0	4
	Vertebrate	0	3
	Xylocopa sp.	6	6

Supplementary Table 3 – Summary table of hierarchical meta-analysis models for (a) pollination service and (b) pollination deficit. Table shows total heterogeneity ("Total"- only effects of pollination service or deficit) and between-group heterogeneity explained by moderators, with corresponding residual heterogeneity in brackets. The table reports two-sided p-values.

	**(a) Pollination service**			**(b) Pollination deficit**		
	**df**	**Q**	***p***	**df**	**Q**	***p***
Total	1196	94985.2	<0.001	681	29894.9	<0.001
Quality trait, 2 levels (residual)	1 (1195)	63.4 (83014.5)	<0.001 (<0.001)	1 (680)	24.2 (29176.3)	<0.001 (<0.001)
Quality trait, 7 levels (residual)	6 (1190)	93.4 (67113.9)	<0.001 (<0.001)	6 (675)	26.6 (28719.6)	0.0002 (<0.001)
Pollinator group (residual)	3 (1193)	3.6 (94062.6)	0.311 (<0.001)	3 (678)	4.5 (28195.2)	0.216 (<0.001)
Crop type (residual)	5 (1191)	5.6 (94184.5)	0.350 (<0.001)	5 (676)	5.6 (26957.7)	0.353 (<0.001)
Experimental scale (residual)	2 (1183)	2.4 (79312.7)	0.297 (<0.001)	2 (662)	2.1 (26907.3)	0.343 (<0.001)
Cropping environment (residual)	1 (1195)	0.9 (94905.5)	0.339 (<0.001)	1 (680)	0.4 (29059.1)	0.551 (<0.001
Climate (residual)	2 (1194)	0.1 (68948.8)	0.954 (<0.001)	2 (679)	3.0 (29383.0)	0.225 (<0.001)

Supplementary Table 4 – List of quality traits investigated in the studies included in our meta-analysis. The table specifies the quality trait categories used in the analyses, the number of effect sizes per individual crop quality trait and per pollination metric.

Quality trait categories used in the meta-analysis (I)	Quality trait categories used in the meta-analysis (II)	Original quality trait measured in the single studies	N effect sizes pollination deficit	N effect sizes pollination service
Organoleptic	Commercial grade	Proportion of "extra quality" fruits	0	3
		Proportion of marketable fruits	3	15
		Percentage of healthy fruits	0	2
		Shelf life	24	15
	Firmness	Firmness/Hardness	23	40
	External appearance and taste ("sensory")	Brightness	7	16
		Colour	12	23
		Sensory perception (e.g., taste)	5	1
	Shape	Proportion of regularly shaped fruits	11	41
		Roundness	21	3
		Dimensions-based size index	0	2
	Size	Circumference	5	57
		Diameter	78	111
		Height, length	52	133
		Thickness	13	14
		Volume	20	9
		Weight	191	334
		Width	29	37
Nutritional	Macronutrients	Carbohydrates	4	2
		Fiber	0	3
		Juice content	4	2
		Oils and fats	19	59
		Protein	2	12
		Pulp content	0	1
		Pulp:seed ratio	3	0
		Sugar:acid ratio	8	19
		Sugars and soluble solids	94	90
		Water content	2	3
	Micronutrients	Acids	24	28
		Ash and dry matter	7	13
		Polyphenols	6	81
		Minerals	6	8
		pH	7	14
		Vitamins	2	6

Supplementary Table 5 – Summary of the random effects tested to identify the best null models. The optimal random effect structure was identified using AIC, the lowest being highlighted in bold for each dataset. All null models contain a variance-covariance matrix assuming 0.5 correlation of sampling errors between effect sizes sharing a common cluster ID.

**Random effects**	**Pollination service null model (AIC)**	**Pollination deficit null model (AIC)**
~1|publication, ~1|individual effect size (residual)	361.07	**-334.74**
~1|publication, ~1|country, ~1|individual effect size (residual)	360.55	-332.74
~1|publication/experimental year, ~1|individual effect size (residual)	358.72	-332.74
~1|country/publication, ~1|individual effect size (residual)	360.16	-330.48
~1|publication, ~1|experimental year, ~1|individual effect size (residual)	**352.50**	-332.74

Supplementary Table 6 – Results of the Egger's regression models testing for funnel plot asymmetry. Slope estimates (95% CI), p-values and marginal R^2 for Egger's regression models including square-root of the inverse of the effective sample size and quality trait for the pollination service and pollination deficit datasets. The table reports two-sided p-values (null hypothesis: slope=0).

Metric	Estimate (95% CI)	p-value	R^2
Pollination service	0.119 (-0.009 : 0.248)	0.068	0.100
Pollination deficit	-0.081 (-0.203 : 0.041)	0.192	0.043

Supplementary Table 7 – Results of the sensitivity analyses on overall pollination service and deficit effects. Sensitivity analyses were performed comparing a) the original models included in the main text with b) models leaving out studies providing more than 5% of the effect sizes (sensitivity model 1), with c) models leaving out influential studies detected with Cook's distance (sensitivity model 2), with d) models assuming a correlation of 0.8 between sampling errors and effect sizes (sensitivity model 3), and with e) models clustering effect sizes at the level of individual studies in the construction of variance-covariance matrices (sensitivity model 4).

**Model**	**Pollination service (estimate and 95% CI)**	**Pollination deficit (estimate and 95% CI)**
a) Original model	22.5% (15.9% : 29.5%)	1.5% (-1.8% : 4.9%)
b) Sensitivity model 1	22.5% (15.7% : 29.8%)	1.7% (-1.7% : 5.2%)
c) Sensitivity model 2	20.3% (14.1% : 26.8%)	0.1% (-2.0% : 2.3%)
d) Sensitivity model 3	22.2% (15.4% : 29.3%)	1.2% (-1.9% : 4.5%)
e) Sensitivity model 4	20.5% (14.4% : 26.9%)	1.2% (-2.0% : 4.5%)

Supplementary Note

Information on the R software environment used in the analyses.

R version 4.2.2 (2022-10-31 ucrt)

Platform: x86_64-w64-mingw32/x64 (64-bit)

Running under: Windows 10 x64 (build 22621)

Matrix products: default

locale: LC_COLLATE=Italian_Italy.utf8, LC_CTYPE=Italian_Italy.utf8, LC_MONETARY=Italian_Italy.utf8 LC_NUMERIC=C,LC_TIME=Italian_Italy.utf8

attached base packages: grid, stats, graphics, grDevices, utils, datasets, methods, base

other attached packages: ggplot2 (v.3.4.1), metaAidR (v.0.0.0.9000), orchaRd (v.2.0), matrixcalc (v.1.0-6), metafor (v.4.2-0), numDeriv (v.2016.8-1.1), metadat (v.1.2-0), Matrix (v.1.5-1), vcd (v.1.4-11)

loaded via a namespace (and not attached): pkgload (v.1.3.2), splines (v.4.2.2), shiny (v.1.7.4), vipor (v.0.4.5), remotes (v.2.4.2), sessioninfo (v.1.2.2), pillar (v.1.8.1), lattice (v.0.20-45), glue (v.1.6.2), digest (v.0.6.31), promises (v.1.2.0.1), colorspace (v.2.0-3), sandwich (v.3.0-2), cowplot (v.1.1.1), htmltools (v.0.5.4), httpuv (v.1.6.9), pkgconfig (v.2.0.3), devtools (v.2.4.5), purrr (v.1.0.1), xtable (v.1.8-4), mvtnorm (v.1.1-3), scales (v.1.2.1), processx (v.3.8.0), later (v.1.3.0), emmeans (v.1.8.4-1), tibble (v.3.1.8), mgcv (v.1.8-41), farver (v.2.1.1), generics (v.0.1.3), usethis (v.2.1.6), ellipsis (v.0.3.2), TH.data (v.1.1-1), cachem (v.1.0.7), pacman (v.0.5.1), withr (v.2.5.0), cli (v.3.6.0), survival (v.3.4-0), magrittr (v.2.0.3), crayon (v.1.5.2), mime (v.0.12), memoise (v.2.0.1), estimability (v.1.4.1), ps (v.1.7.2), fs (v.1.5.2), fansi (v.1.0.3), nlme (v.3.1-160), MASS (v.7.3-58.1), beeswarm (v.0.4.0), pkgbuild (v.1.4.0), profvis (v.0.3.7), tools (v.4.2.2), prettyunits (v.1.1.1), lifecycle (v.1.0.3), multcomp (v.1.4-22), stringr (v.1.5.0), munsell (v.0.5.0), callr (v.3.7.3), compiler (v.4.2.2), rlang (v.1.1.0), rstudioapi (v.0.14), htmlwidgets (v.1.6.1), miniUI (v.0.1.1.1), labeling (v.0.4.2), codetools (v.0.2-18), gtable (v.0.3.1), DBI (v.1.1.3), R6 (v.2.5.1), zoo (v.1.8-11), dplyr (v.1.0.10), fastmap (v.1.1.1), utf8 (v.1.2.2), mathjaxr (v.1.6-0), latex2exp (v.0.9.6), ggbeeswarm (v.0.7.2), stringi (v.1.7.12), Rcpp (v.1.0.10), vctrs (v.0.6.1), tidyselect (v.1.2.0), urlchecker (v.1.0.1), coda (v.0.19-4), lmtest (v.0.9-40)

Supplementary References

1. Nakagawa, S. et al. orchaRd 2.0: An R package for visualising meta-analyses with orchard plots. *Methods Ecol. Evol*, 1-8 (2023).
2. Peters, J. L. et al. Contour-enhanced meta-analysis funnel plots help distinguish publication bias from other causes of asymmetry. *J. Clin. Epidemiol.* **61**, 991-996 (2008).
3. Klein, A. M. et al. Importance of pollinators in changing landscapes for world crops. *Proc. R. Soc. B Biol. Sci.* **274**, 303–313 (2007).
