# Supplementary material for: Global meta-analysis shows reduced quality of food crops under inadequate animal pollination
Source: Nat Commun. 2023 Jul 25;14:4463. doi: 10.1038/s41467-023-40231-y (PMC10368628; doi:10.1038/s41467-023-40231-y)
Supplement: Supplementary file 3 — Description of Additional Supplementary Information [file 41467_2023_40231_MOESM3_ESM.docx]

**Description of Additional Supplementary Files**

**Global meta-analysis shows reduced quality of food crops under inadequate animal pollination**

**Authors:**

Elena Gazzea^1^*, Péter Batáry^2^, Lorenzo Marini^1^

**Affiliations:**

^1^ Department of Agronomy, Food, Natural resources, Animals and Environment (DAFNAE), University of Padua, Legnaro (Padua), Italy

^2^ “Lendület” Landscape and Conservation Ecology, Institute of Ecology and Botany, Centre for Ecological Research, Vácrátót, Hungary

* Corresponding author, e-mail: elena.gazzea@unipd.it

**File Name:** Supplementary Data 1
**Description:** Preferred Reporting Items for Systematic Reviews and Meta-Analyses for Ecology and Evolutionary Biology (PRISMA EcoEvo) checklist.

**File Name:** Supplementary Data 2
**Description:** List of publications used in the meta-analysis.
