# Supplementary material for: Global meta-analysis shows reduced quality of food crops under inadequate animal pollination
Source: Nat Commun. 2023 Jul 25;14:4463. doi: 10.1038/s41467-023-40231-y (PMC10368628; doi:10.1038/s41467-023-40231-y)
Supplement: Supplementary file 6 — Reporting Summary [file 41467_2023_40231_MOESM6_ESM.pdf]

nature portfolio

Corresponding author(s): Elena Gazzea

Last updated by author(s): Jul 4, 2023

# Reporting Summary

Nature Portfolio wishes to improve the reproducibility of the work that we publish. This form provides structure for consistency and transparency in reporting. For further information on Nature Portfolio policies, see our Editorial Policies and the Editorial Policy Checklist.

## Statistics

For all statistical analyses, confirm that the following items are present in the figure legend, table legend, main text, or Methods section.

| n/a | Confirmed | |
|---|---|---|
| ☐ | ☒ | The exact sample size (*n*) for each experimental group/condition, given as a discrete number and unit of measurement |
| ☐ | ☒ | A statement on whether measurements were taken from distinct samples or whether the same sample was measured repeatedly |
| ☐ | ☒ | The statistical test(s) used AND whether they are one- or two-sided<br>*Only common tests should be described solely by name; describe more complex techniques in the Methods section.* |
| ☐ | ☒ | A description of all covariates tested |
| ☐ | ☒ | A description of any assumptions or corrections, such as tests of normality and adjustment for multiple comparisons |
| ☐ | ☒ | A full description of the statistical parameters including central tendency (e.g. means) or other basic estimates (e.g. regression coefficient) AND variation (e.g. standard deviation) or associated estimates of uncertainty (e.g. confidence intervals) |
| ☐ | ☒ | For null hypothesis testing, the test statistic (e.g. *F*, *t*, *r*) with confidence intervals, effect sizes, degrees of freedom and *P* value noted<br>*Give P values as exact values whenever suitable.* |
| ☒ | ☐ | For Bayesian analysis, information on the choice of priors and Markov chain Monte Carlo settings |
| ☐ | ☒ | For hierarchical and complex designs, identification of the appropriate level for tests and full reporting of outcomes |
| ☐ | ☒ | Estimates of effect sizes (e.g. Cohen's *d*, Pearson's *r*), indicating how they were calculated |

*Our web collection on statistics for biologists contains articles on many of the points above.*

## Software and code

Policy information about availability of computer code

| | |
|---|---|
| Data collection | Literature survey was conducted in Scopus, ISI Web of Science, and Google Scholar. Data were extracted from figures through WebPlotDigitizer (version 4.6) and metaDigitise (version 1.0.1) R package. Data was structured and coded using Microsoft Excel spreadsheet. |
| Data analysis | All analyses were conducted using the package metafor (version 4.2-0) in R software (version 4.2.2). Collinearity among tested moderators were visualised using the package vcd (version 1.4-11). See Supplementary Note for detailed information on the R software environment used in the analyses. |

For manuscripts utilizing custom algorithms or software that are central to the research but not yet described in published literature, software must be made available to editors and reviewers. We strongly encourage code deposition in a community repository (e.g. GitHub). See the Nature Portfolio guidelines for submitting code & software for further information.

## Data

Policy information about availability of data

All manuscripts must include a data availability statement. This statement should provide the following information, where applicable:

- Accession codes, unique identifiers, or web links for publicly available datasets
- A description of any restrictions on data availability
- For clinical datasets or third party data, please ensure that the statement adheres to our policy

The data and the code used in this study have been deposited on Zenodo digital repository under https://doi.org/10.5281/zenodo.8113788. Literature search databases used in this study are: ISI Web of Science (WoS) Core Collection; Scopus; Google Scholar.

## Research involving human participants, their data, or biological material

Policy information about studies with human participants or human data. See also policy information about sex, gender (identity/presentation), and sexual orientation and race, ethnicity and racism.

| | |
|---|---|
| Reporting on sex and gender | Not applicable. |
| Reporting on race, ethnicity, or other socially relevant groupings | Not applicable. |
| Population characteristics | Not applicable. |
| Recruitment | Not applicable. |
| Ethics oversight | Not applicable. |

Note that full information on the approval of the study protocol must also be provided in the manuscript.

# Field-specific reporting

Please select the one below that is the best fit for your research. If you are not sure, read the appropriate sections before making your selection.

☐ Life sciences ☐ Behavioural & social sciences ☒ Ecological, evolutionary & environmental sciences

For a reference copy of the document with all sections, see nature.com/documents/nr-reporting-summary-flat.pdf

# Ecological, evolutionary & environmental sciences study design

All studies must disclose on these points even when the disclosure is negative.

| | |
|---|---|
| Study description | We conducted a meta-analysis to quantify the contribution of pollination to food crop quality. We used hierarchical multi-level meta-analysis models to examine the effect of pollination on a wide range of organoleptic and nutritional quality traits across crops important in human diet. We tested the effect of both pollination service (i.e. difference between pollinators exclusion and open pollination) and pollination deficit (i.e. difference between open pollination and hand pollination) on food crop quality. We quantified the effect of pollination service and deficit using the natural logarithm of the response ratio between experimental treatment means as a measure of effect size. We used 1197 effect sizes for pollination service and 682 effect sizes for pollination deficit analyses. We used moderators to explore the heterogeneity of effect sizes for both pollination service and deficit. We tested the following moderators: quality trait, pollinator group, crop type, experimental scale, cropping environment, climate, and year of publication. We did not test for interactions among moderators. |
| Research sample | We followed the PRISMA protocol for study selection and inclusion in the systematic review and meta-analysis. Quantitative analyses were performed for 1197 effect sizes from 153 studies for pollination service, and 682 effect sizes from 86 studies for pollination deficit. See Methods for a definition of the pollination metrics used. |
| Sampling strategy | We conducted two literature searches, a first search in 2021 and an updated search in 2023. The literature was searched through ISI Web of Science Core Collection, Elsevier Scopus, and Google Scholar. We followed the PRISMA protocol for study selection and inclusion in the systematic review and meta-analysis. |
| Data collection | Elena Gazzea conducted the literature search, screened all literature, extracted all the effect sizes, and coded the information from the studies. |
| Timing and spatial scale | A first literature search was conducted in January 2021. Following peer-review process, we updated our search string, and we conducted another literature search in February 2023. The included studies were published between 1968 and 2023. Year and geographical area of the studies are reported in Supplementary Fig.1 and Figure 2, respectively. |
| Data exclusions | Identified potentially relevant literature was assessed against predefined inclusion criteria. First, we only included studies reporting results derived from primary data of manipulative experiments. Second, we included studies measuring at least one quality trait (see |

| | |
|---|---|
| | Supplementary Table 4 for a list of quality traits included). We excluded publications reporting solely fruit set, yield, seed germination, seed set, or seed number. Third, experiments included at least two different pollination treatments, so one of the pollination metrics of interest could be calculated (see Figure 1 and Methods for a definition of the pollination metrics used). Finally, we excluded experiments when fruit production was insufficient to assess quality. Studies with missing, non-retrievable data (mean, standard deviation, number of replicates) were also excluded. We did not exclude any of the extracted effect sizes from main analysis. |
| Reproducibility | The methods of data collection and analysis are presented in the Methods section in detail. Search strings and selection process of both initial (January 2021) and updated (to February 2023) literature surveys are reported. All data and code have been deposited on Zenodo under https://doi.org/10.5281/zenodo.8113788. |
| Randomization | Not applicable - this is a meta-analysis based study. |
| Blinding | Not applicable - this is a meta-analysis based study. |

Did the study involve field work? ☐ Yes ☒ No

# Reporting for specific materials, systems and methods

We require information from authors about some types of materials, experimental systems and methods used in many studies. Here, indicate whether each material, system or method listed is relevant to your study. If you are not sure if a list item applies to your research, read the appropriate section before selecting a response.

## Materials & experimental systems

| n/a | Involved in the study |
|---|---|
| ☒ | ☐ Antibodies |
| ☒ | ☐ Eukaryotic cell lines |
| ☒ | ☐ Palaeontology and archaeology |
| ☒ | ☐ Animals and other organisms |
| ☒ | ☐ Clinical data |
| ☒ | ☐ Dual use research of concern |
| ☒ | ☐ Plants |

## Methods

| n/a | Involved in the study |
|---|---|
| ☒ | ☐ ChIP-seq |
| ☒ | ☐ Flow cytometry |
| ☒ | ☐ MRI-based neuroimaging |
